# Effectiveness of psychosocial intervention for teenage pregnancy on low birth weight and preterm birth outcomes: a systematic review and meta-analysis

**DOI:** 10.1186/1472-6963-14-S2-P120

**Published:** 2014-07-07

**Authors:** Kanokporn Sukhato, Chathaya Wongrathanandha, Pornpoj Horsuwansak, Thunyarat Anothaisintawee

**Affiliations:** 1 Department of Family Medicine, Faculty of Medicine, Ramathibodi Hospital, Mahidol University, Bangkok, Thailand

## Background

Teenage pregnant women are at risk of having low birth weight and preterm birth babies. The additional psychosocial interventions are thought to improve these outcomes for teenage pregnancy. However, the effect of additional psychosocial intervention programs on those is uncertain. The aim of this meta-analysis is to assess the effects of additional psychosocial interventions for teenage pregnancy on low birth weight and preterm birth outcomes.

## Methods

*Search strategy:* Relevant studies were identified from Medline, CINAHL, and Scopus since initiation to November 2013.

*Selection criteria:* Randomized controlled trials investigating the effect of any psychosocial intervention on low birth weight and preterm birth were included.

*Data collection and analysis:* Two reviewers independently assessed the quality of each study and extracted outcomes including rate of low birth weight, preterm birth rate, mean gestational age at delivery and mean birth weight. Standardized mean difference and DerSimonian-Laird method were applied for pooling continuous and dichotomous outcomes, respectively.

## Results

The five studies with 712 teenage pregnant women were included. Compared with routine antenatal care, the psychosocial intervention significantly reduced risk of low birth weight by 40% (pooled RR=0.60, 95% CI: 0.38, 0.92). For the preterm birth, the psychosocial intervention reduced risk of preterm birth by 33% with no statistical significance (pooled RR=0.67, 95% CI: 0.42, 1.05). The pooled result for birth weight showed that our infants in the intervention group are slightly heavier than those of control group with statistical significance (WMD 200.63, 95% CI: 21.02, 380.25). Gestational age at delivery of intervention group was slightly increased than those of control group with no statistical significance (WMD 0.293, 95% CI:-0.43, 1.02).

## Conclusion

The additional psychosocial support to teenage pregnant women can improve pregnancy outcomes including low birth weight and birth weight. The variation in the intervention and risk of bias within included studies may limit this conclusion.

